# Deprivation, discrimination, human rights violation, and mental health of the deprived

**DOI:** 10.4103/0019-5545.70972

**Published:** 2010

**Authors:** R. C. Jiloha

**Affiliations:** Department of Psychiatry, GB Pant Hospital, Maulana Azad Medical College and Faculty of Medical Sciences, University of Delhi, Delhi, India

## INTRODUCTION

Human behavior is conceived of as an outcome of genetic and biochemical characteristics, past learning experiences, motivational states, psycho-social antecedents, and the cultural context in which it unfolds[1] Culture plays a complex role in the natural history and psycho-social development of human behavior[2] comprising of customs, beliefs, values, knowledge, and skills.[3] Social norms, the shared rules that specify appropriate and inappropriate behaviors;[4] mores, that people consider vital to their well-being and to their most cherished values,[5] and sanctions, the socially imposed rewards and punishments that compel people to comply with norms,[6] constitute important ingredients of a culture. Orlandi *et al*. (1992),[2] define culture as shared values, beliefs, norms, traditions, customs, art, history, folklore, and institutions of a group of people. A society which is a cohesive group of people shares all the ingredients of the culture among its members.

The Indian subcontinent has been likened to a deep net into which various races and people have drifted and been caught in the remote past and their diverse origins have dictated variety. Geographical conditions of the sub-continent forced these varied people to stay together in a multiple society imposing on them what has been described by historians as ‘Unity in Diversity’ having cultural homogeneity.[7]

## UNITY IN DIVERSITY OR ETHNIC DIVERSITY

Within the perspective and definition of culture, Indian society as a social group does not have one culture, because this society consists of several racial, ethnic, religious, and caste groups that have their own beliefs, customs, values, art, history, and folklore, and identify with their respective groups.[8] As one social group, therefore, Indian society has no cultural homogeneity contrary to what historians describe.

The concept of ‘Unity in Diversity,’ which means complete harmony, peace, and adjustment among different cultural elements arises from a view of accepting the textual position of the Hindu social system, and explaining as well as justifying the empirical social reality in terms of the Vedic scriptures,[9] based on a consensus model. The concept of ’Unity in Diversity’ gives rise to an erroneous understanding of India’s social reality.

Contrary to ‘Unity in Diversity’ there is ethnic diversity in the Indian society, leading to the formation of minority groups who differ from majority prototype not only in terms of numerical strength of their members, but also in their access to various resources. As the societies are governed by their members, it is obvious that the majority group will have the maximum say.[6] Therefore, there has been a dialectical interrelationship between majority social groups who have monopolized the scarce goods of power and prestige and minorities those who lack these resources. Deprivation plays an important role in the unfolding of human behavior in Indian society.[8] Ethnic minorities are most subjected to being at receiving end of social deprivation, particularly when religious scriptures and social sanctions permit the deprivations.[10]

Deprivation is the consequence of socioeconomic disparity due to the caste-system that is peculiarly fitted in the Indian society; to hand on cultural patterns and particular items of the culture. The traditional Hindu society that is compartmentalized into various caste-groups is a social institution dictating superior and lesser beings among its members. This system that places the untouchables at the bottom of the caste-pyramid is one of the obvious institutions of caste-inequality, a system of legalized inequality, a variant of an ascriptive system of stratification, wherein, the allocation of roles and status is governed by its own principles, determining the social, economic, political, and ritualistic structure of individuals in relation to each other.[11]

The essence of the caste is the arrangement of hereditary groups in a hierarchy, as a necessary corollary the caste confines the individual in the occupation handed down from father to the son and governed by precise rules regarding the acceptance or rejection of food or water from the members of the other caste.[12] Caste-system in India has had its impact on all aspects of life; on the past, present, and future, based on purity and pollution basis. Birth only determines the individual’s social status throughout his life[13] and also his access to various resources.

The deprived masses described compendiously as Scheduled castes and Scheduled tribes in the constitution of India are in fact low castes and tribes in the Hindu social order, treated as ‘caste-less’, outcastes or untouchables and have been subjected to deprivation and discrimination for centuries.[14] Historically, they spring from the aboriginal inhabitants, conquered and enslaved by Aryan invaders.

For the first time in history, untouchables were accorded equal status to other citizens in the constitution of independent India. With the desire to bring them in step with the privileged ones, the policy of reservations was introduced, offering them the advantage of education and jobs.[15] The last 62 years of independence have witnessed a massive social mobility and transformation as well as the emergence of ’the educated’ among the deprived castes, generally looked down upon with contempt by the larger society for their mobility on the crutches of reservations.[16] Although untouchability is outlawed and the caste-system is not overtly practiced, at least in the bigger cities, there are other ways of isolating and segregating them similar to abolition of slavery in the USA, where injustice to African-Americans continued until the passing of the Civil Rights Act.[17] Emergence of the ’educated among the deprived’ and their journey from traditional defiling occupations to white-collared respectable office jobs has created an environment, exposing them to various psychological and physical vulnerabilities causing mental health strains.

## HEALTH AS A HUMAN RIGHT

The preamble of the World Health Organization (WHO) succinctly underscores the enjoyment of the highest standard of health as a fundamental right of every human being. According to Article 25 of the Universal Declarations of Human Rights, every one has the right to a standard of living, adequate for the health of himself, including food, clothing, housing, medical care, and necessary services. Studies reveal that individuals’ poorer health status, including higher morbidity, lower life expectancy, and higher rates of infant mortality are linked to their race, ethnicity, and caste. Studies also reveal that any kind of discrimination rooted in social, including caste or racial origin affects people’s health in at least three distinct ways: (a) health status, (b) access to healthcare, and (c) in quality of health services.[18] Health is defined as a state of complete physical, mental, and social well-being and not merely the absence of disease or infirmity.[19] It is a basic and dynamic force in our daily lives, influenced by our circumstances, beliefs, and culture, and social, economic, and physical environment. Health has also been defined in the following words:

Health is a unity and harmony within the mind, body, and spirit, which is unique to each person. The level of wellness or health is, in part, determined by the ability to deal with and defend against stress. Health is on a continuum with movements between a state of optimum well-being and illness. It is determined by physiological, psychological, socio-cultural, spiritual, and developmental stage variables.[16]

Mental Health is described as an appropriate balance between the individual, his social group, and the larger environment. These three components combine to promote psychological and social harmony, a sense of well being, self-actualization, and environmental mastery.[19]

In the Indian context, where social diversity, stratification, reservations, social mobility, contempt, deprivation, discrimination prejudice, rejection and socio-technological change are operating in such a complex manner, mental health assumes great significance. Mental heath is not simply the absence of mental illness, but a positive concept of displaying an ability to adapt to social and interpersonal relationships and to reach a harmonious relationship with the society.[10] It is the mental health component of overall health that gives quality and meaning to our lives.[20] When the individual is unable to cope with the changes, it not only affects his social role, but also disturbs the psycho-social homeostasis.[21] As such ethnic minorities are subjected to mental health strains[10] the subject assumes greater importance when these changes operate in a diverse and complex manner to influence human behavior in Indian society.

## MENTAL HEALTH ISSUES IN DEPRIVED CASTES

### Deprivation and psychosocial development

The early developmental process is considered to be largely epigenetic, but the biological material blossoms only in the psycho-social milieu, which has its influence on the development.[10] In a study, at the age level of four-to-five years, the performance scores of privileged and deprived caste children have been similar, but with advancing age and years of schooling the difference accentuated in favor of the privileged caste children producing a ‘torch light effect’.[22] A child born in a traditionally deprived-caste, poor family, suffers from protein-calorie malnutrition, which may retard his intellectual growth. It is evident from Table 1 that the ‘Children belonging to Scheduled castes, Scheduled tribes, and Other Backward Castes’ have higher levels of under nutrition compared to the national average, according to all three measures, namely, (i) weight-for-age, (ii) height-for-age, and (iii) weight-for-height[23][Table 1].

**Table 1:** Percentage of three-year-old children classified as undernourished on three anthropometric indices of nutritional status, India, 1998–1999

Castes/Communities	Weight-for-age % below		Height-for-age % below		Weight-for-height % below	
	
	-3 SD	-2 SD	-3 SD	-2 SD	-3 SD	-2 SD
SCs	21.2	53.5	27.5	51.7	3.0	16.0
STs	26.0	55.9	27.6	52.8	4.4	21.8
OBCs	18.3	47.3	23.1	44.8	3.4	16.6
Others	13.8	41.1	19.4	40.7	1.8	12.8
India	18.0	47.0	23.0	45.5	2.8	15.5

Signify SD

Source: The National Family Health Survey (NFHS-2), 1998–1999, IIPS, and ORC Macro, 2000; p. 269.[23]

Early environmental influences of stimulation or isolation have a marked effect on measurable cognitive functions.[24] Social class differences affecting language development emerge during the first year of life. Early environmental influences have a marked effect on the measurable functioning of vocabulary,[25] and social class differences affecting the language development become unequivocal by three years of age.[26] Some amount of linguistic deficiency due to this socio-cultural disadvantage has been observed in rural children also, as compared to urban ones.[17] Language is an important weapon of expression and shapes an individual’s personality, which the deprived caste children lack in comparison to the privileged ones. Deprivation from somato-sensory stimulation leads to inadequate intellectual growth. Between the ages of three and six years, incipient attitudes of his/her social group show systematic development, which gets correctly categorized around eight years of age.[27] A child growing in a disadvantaged family may suffer on account of not being able to enter the larger mainstream of society, as his social circle remains sharply limited.[10] Such a child would emerge out with a checkered personality, ill-equipped to face the divergent ways of society and culture at large.[24]

## SCHOLASTIC DIFFICULTIES IN DEPRIVED CHILDREN

Human resources are essential components for human development and education is given overriding the priority, to achieve the goal. The process of education begins in the family where the child spends most of his time and receives informal learning, which gradually prepares him for the formal education.[28] Among a number of equalities offered to deprived castes, equality in education is enshrined in the constitution of free India.[15] However, the spread of education among the deprived castes has remained very slow due to various reasons.[16] For several decades, low achievement among children of deprived castes has been a serious problem. The gap in educational achievement in these children and children from privileged castes increases significantly during the elementary and secondary grades. This gap can be primarily attributed to, (a) inadequate educational facilities (b) lack of motivation, and (c) socio-economic status of parents.[27] Parental illiteracy, low economic status, large size of the family, and impoverished home environment are the contributing factors for low educational achievement.[29–32]

Poverty and ignorance were the main hindering factors, especially during the first three to four decades of independence.[16,32] In the initial years, high dropout rate from the schools was observed among deprived caste students, as they were not tolerated by the privileged caste students for their unclean status, while later when these students started making their presence felt in the educational institutions in increasing numbers, they became a source of irritation, heart-burn, and inter-community tension.[22]

Capacity to respond to and benefit from education depends upon a child’s intellect, language, and emotional maturity. As the children from the traditionally deprived communities lack a role model to follow in the area of education, they carry low aspiration for money, material things, and occupational status.[33] It is observed that they have very low self-concept, low self-esteem, and lower need to achieve. They have an overwhelming concern for the immediate needs of sustenance, with a static level of aspirations. They are extremely cautious and avoid taking risks, where a possibility of failure is present.[22] They are generally vague, fantasy-oriented in their future planning, comparatively older in age, and from illiterate parents.[34]

India’s deprived caste illiterate parents engaged in defiling and hateful occupations, breed emotionally less stable children. Due to lack of a meaningful interaction in early childhood, they have poor verbal language expression and lack of stimulation makes their intellect sluggish. Destined to low achievement because of lack of qualitative interaction and inadequate development of cognitive and linguistic skills, they lag behind in their scholastic achievements.

## REACTION TO DISCRIMINATION, REJECTION

Human-beings are acutely responsive to how and what other people perceive, evaluate, and feel about them.[35,36] Positive and negative reactions from others often affect the quality of interpersonal relationship.[37] Behavioral scientists have documented that positive responses from others foster a psychological and physical well-being, whereas, long-term exposure to negative reactions is associated with psychological difficulties and poor physical health.[38,39]

People who experience rejection generally have three sets of motives. The first motive involves a heightened desire for social connections, those who can possibly provide acceptance and support; the second set of motives involves angry, antisocial urges to defend oneself or to hurt the source of rejection; third, the rejected people are motivated to avoid further rejection,[40] therefore withdraw themselves. Members of India’s deprived castes often crave for establishing social connections with the members of privileged castes and wish to gain their acceptance, and when they fail in doing so anger and hostility generates. Anger and aggression are common responses to rejection and often lead to long-lasting break in social bonds.[41] Studies have shown aggressive behavior among rejected school children by their peer group.[42] Anger and aggression in rejected children occurs as a result of pain or frustration associated with rejection.[43] Moreover, rejection by the peer group not only creates a great deal of suffering in the child, but also predicts negative emotional and behavioral outcomes in the future.[44]

Rejected people may withdraw from and avoid interpersonal interactions, not only with those who rejected them but often with other people as well. They may either physically leave the situation or withdraw socially and psychologically, while remaining physically present when they cannot escape or avoid social encounter.[45] The events that connote rejection immediately elicit negative emotions, such as, sadness, loneliness, hurt, anger, jealousy,[41] and lower self-esteem in the victims. India’s deprived castes who perceive rejection from the majority groups breed negative emotions, low self-esteem, avoidance behavior, and aggressive traits among their members.

## STIGMATIZATION

Social stigma refers to a ‘defect’ in a person’s social identity-negative information about a person that is known by others. In the traditional Hindu social hierarchy an untouchable is evaluated so low that the depth of degradation accords him a sub-human status.[13] Negative reactions from others may take many forms – ranging from disinterest, criticism, prejudice, avoidance, rejection, betrayal, stigmatization, ostracism, abandonment, and abuse to bullying.[35] On account of the stigmatized existence, a deprived caste student is highly self-conscious, sensitive to others’ comments and criticism, has real or imagined evaluation, and is likely to feel socially anxious, especially when under observation.[36] The psychological core of all instances is the stigma in which a person is the recipient of negative reactions.

## SOCIAL CHANGE AND INDIVIDUAL ROLE

A role is the part that a person plays within the given social context.[46] Associated with each role is a set of expectations regarding the appropriate behavior of the occupant for that role. A stable society has clear role definitions, while the social change burdens the individual with new role demands. Role novelty occurs when a person finds himself in a position he has not previously occupied and while playing a new role, he may be unaware of which behavior he perceives to be appropriate.[47] This is a common source of one’s uncertainty about self-presentation and triggers social anxiety.[48]

In the traditional Hindu society everyone has an explicitly defined social role. Members of the erstwhile low castes were assigned the role of serving the members of higher castes. With the advent of modern education, urbanization, and new technologies, there has been a massive occupational mobility from the traditionally hateful and defiling occupations to the newly created respectable white-collar positions, the first-generation educated deprived finds him in a new role with many psychological difficulties. Motivation for change is an important factor for altering behavior pattern and a person is more likely to adjust to the change if he perceives the change to be desirable.

## PERSONALITY PROFILE OF DEPRIVED-CASTE STUDENTS

The university and professional college students hailing from illiterate deprived caste families generally do not attribute their success to their own efforts and hard work, rather they refer their success to external factors such as the kindness of their teachers, mercy of God, and their good luck.[16] This tendency leads to superstitious behavior, perpetuation of a fatalistic outlook, ritualism, and ingratiation of their significant others. On the other hand, failure is often ascribed to oneself. These students have harsh self-criticism, less favorable self-concept, and rigid standards to evaluate one’s own performance.[24] It builds an ego-damaging and self-discouraging internalized mechanism.

In a study of medical students of various categories, it was found that the deprived students had low activity and cyclothymic temperament. Depression and emotional instability was observed to be higher in these students. They exhibited more of the socially desirable behavior than others.[49]

‘Cognitive approach hypothesis’, says that negative self-evaluation results in social anxiety. This anxiety leads to avoidance behavior in certain social situations that demand their attention and decision making.[50] In these situations they are found to be more tense and anxious for the fear of things going wrong.[51] A deprived-caste student is generally more cautious, careful, and guarded as compared to the privileged caste student.[52]

‘Memory for self-relevant information’ research suggests that once a person views himself and his performance negatively, he is more likely to recall incidents in which he performed poorly. These easily accessed negative memories serve to precipitate social anxiety when future encounters are contemplated.[13]

People are generally sensitive to others perception and evaluation about them[36] and they are highly motivated to seek others’ approval, acceptance, and affection, than to seek others disapproval and rejection. When need for approval is high, a person tries to manage a better impression. Therefore, the factors that heighten people’s motivation to seek approval are associated with increased social anxiety and that is why a person’s feelings of self-worth are partially dependent on other’s evaluation.[53] Deprived caste students who are in want of social approval and acceptance, carry high levels of social anxiety as compared to the general population of students. This anxiety interferes with their work efficiency resulting in their poor performance.

Studies reveal that deprived-caste students have unrealistic motivation, external locus for success, personal inadequacies for failures, harsh and rigid self-evaluation, and extreme anxiety for the outcome of personal performance. They exhibit avoidance behavior, lack the decision-making capacity, tend to have negative memories of past experiences, carry a very low self-concept, and need social approval. Success is not that reinforcing as it should be, and failure is extremely discouraging. They experience the fear of failure because of internalization of personal inadequacies, negative memories, and low perception of self, and heightened social anxiety. The entire mechanism is motivationally damaging and that is why deprived-caste students account for the largest population of failures in examinations and drop-outs from educational institutions.[22,54,55]

India’s deprived castes present a number of difficulties related to mental health. Their developmental process to assume psychological maturity and to achieve mental health is retarded due to factors like deprivation of childhood experiences, lack of qualitative interaction for healthy cognitive and linguistic development, unrealistic motivation, external locus for success, harsh and rigid self-evaluation, high levels of social anxiety, avoidance behavior, and so on. They are exposed to multiple psychological strains that lead to mental health aberrations. Nearly 90 percent of all the poor Indians and 95 percent of all the illiterate Indians are from deprived castes.[56] Thus far, India has not succeeded to uphold its international legal obligations to ensure the fundamental human rights of the deprived, despite laws and policies against caste discrimination.[57]

They need more attention in the form of recognition and encouragement. This need is readily satisfied in privileged group students, while the deprived caste students get far less recognition; yet their need is immeasurably greater.[58] Further research is needed to explore the factors causing their scholastic backwardness and low achievement.
